# Flexural Strength and Radiodensity of Different Barium Sulfate-Containing Polymethyl Methacrylate Provisional Restorative Materials

**DOI:** 10.1055/s-0045-1809978

**Published:** 2025-07-08

**Authors:** Niwut Juntavee, Apa Juntavee, Kesinee Rattanatussanee

**Affiliations:** 1 Department of Prosthodontics, Faculty of Dentistry, Khon Kaen University, Khon Kaen, Thailand; 2 Division of Pediatric Dentistry, Department of Preventive Dentistry, Faculty of Dentistry, Khon Kaen University, Khon Kaen, Thailand; 3 Division of Biomaterials and Prosthodontics, Faculty of Dentistry, Khon Kaen University, Khon Kaen, Thailand

**Keywords:** barium sulfate, CBCT, flexural strength, radiopacity, provisional restoration, radiodensity

## Abstract

**Objectives:**

Provisional restoration fabricated from polymethyl methacrylate (PMMA) faces radiographic clarification due to its radiolucency. This study determined the appropriate radiodensity and strength of auto-polymerized PMMA (P) upon adding barium sulfate (B) at different concentrations.

**Materials and Methods:**

A total of 90 specimens (length × width × height = 30 × 12.5 × 2.0 mm) were prepared from P containing B at different ratios (volume %B): 5P-0B (0%), 4P-1B (20%), 3P-1B (25%), 2P-1B (33.3%), 1P-1B (50%), and 1P-2B (66.7%) ( *n* = 15/group). Specimens were CBCT (cone-beam computed tomography)-scanned and their radiodensity measured in Hounsfield units (HU). A three-point bending test was used to determine flexural strength (σ).

**Statistical Analysis:**

ANOVA (analysis of variance) and Tukey tests were used for analyzing significant differences in radiodensity and σ among groups (α = 0.05). The correlation coefficient ( *R* ) between radiodensity and σ was determined with the Spearman test.

**Results:**

Radiodensity (HU) was intense in 1P-2B (1,080.28 ± 119.8) and softest in 5P-0B (−283.3 ± 73.8), whereas σ (MPa) was highest in 5P-0B (60.3 ± 15.2) and lowest in 1P-2B (15.9 ± 3.4). Significant differences in radiodensity among groups ( *p* < 0.05) were indicated, except between 4P-1B/3P-1B/2P-1B and 2P-1B/1P-1B. Significant differences in σ among groups ( *p* < 0.05) were indicated, except between 3P-1B/2P-1B and 1P-1B/1P-2B. Increasing amount of B intensified radiodensity, but diminished σ, with extreme negative correlation ( *R* = –0.83).

**Conclusion:**

Radiodensity and σ were influenced by the amount of B addition. Raising B intensifies radiopacity but lessens σ. Adding B up to 25% provides adequate radiopacity and strength for provisional restorations for use in high-stress areas. However, adding B ≥33.3% provides better radiographic visibility but minimized strength, suggesting restorations in less-stress areas or other appliances, such as radiographic and surgical guides.

## Introduction

Oral rehabilitation for a patient with partially or fully edentulous arch using implants is a contemporary predictable treatment modality since the implants can support fixed dental restorations to function similarly to their previous natural dentition. The continuously improved techniques to utilize dental implants for oral reconstruction enable the clinician to deliver foreseeable implant-supported restorations with aesthetic and functional achievement. 1 A crucial step that leads to supreme success in implant prosthodontics is the dental implant placement in a proper position in relation to other teeth or implants as well as the plane of occlusion. Appropriate implant positioning is an essential consideration that influences the accomplishment of construction implant-supported restoration. A radiographic guide assisting radiographic imaging provides the precise data needed for the clinician to choose appropriate implant dimensions for placing in an accurate position and angulation. 2 The prosthetically driven implant placement concept was eventually developed due to the need for a predictable prosthesis that encompasses digitally assisted diagnosis, treatment planning, and sequencing of treatment, thus optimizing the biological and restorative foundations of the implant-supported prosthesis. The success of implant treatment is fundamentally associated with comprehensive planning in placing the implant in an accurate position, which is established at the diagnostic phase according to the virtual final restoration. 1 3

Provisional restoration is an essential part of the treatment of implant-supported fixed prosthodontics. It is commonly used as a diagnostic tool for the determination of position, contour, contact, and occlusion of the designed final restoration. It also was used as a guide for radiographic imaging during implant planning, as well as a surgical guide for implant installation. A suitable provisional restoration used as a radiographic and surgical guide should be accurate in dimension, stiff, and stable, which regularly utilizes radiopaque material coated on the provisional restoration. 4 Computed tomography (CT) is fundamentally used for assessing the anatomical structures, bone morphology, and density of bone, which is expressed in the Hounsfield unit (HU). The HU is a relative quantitative scale of radiodensity of material and tissue based on values for air (−1,000, black on the grayscale), water (0), and bone (+700 to +400 for cancellous, and +500 to 1,900 for cortical). 5 The contemporary cone-beam CT (CBCT) shows an extreme linear correlation between the grayscale in CBCT and HU in conventional CT. 6 Nowadays, the assessment of bone quality and bone quantity upon planning for dental implant installation uses the CBCT to assist the clinician in designing for type of dental implant, the implant installation procedure, as well as the surgical technique utilization. 7 The accuracy of implant planning depends on the merging quality between the stereolithography (STL) files derived from the patient's intraoral scanning and the digital imaging and communication in medicine (DICOM) files derived from the CBCT. 8 The provisional restoration used as a radiographic guide per CBCT for planning implant position and being modified to be a surgical guide during the surgery can play an important role in preventing implant placement in undesirable sites, reducing unrequired osteotomy, resulting in appropriate prosthesis design, minimizing the required surgical time and trauma, and favoring patient comfort. 9 An inappropriate implant position and angulation results in a risky surgical procedure, difficulty in restoration construction, and deterioration of the implant prognosis. In severe occlusal plane discrepancy, provisional restoration should be used for creating an ideal occlusal scheme before performing the definitive implant planning. A provisional restoration is also beneficial in the healing of the peri-implant tissue and allows the clinician to conclude for appropriate aesthetic and phonetic amendment. Clinicians frequently use provisional restoration as a guide for shade selection, contouring of a definite restoration, creating soft tissue profile, communicating tools to the dental technicians, and allowing the patient to visualize and evaluate the restoration that might need any modifications required before commencing the conclusive restoration.

Poly-methyl methacrylate [PMMA, (C _5_ O _2_ H _8_ ) _*n*_ ] is generally used for the fabrication of provisional restorations, which can be constructed either using auto-polymerized PMMA at the chair side or energy-cured PMMA in the laboratory. 10 11 12 PMMA is normally presented in a powder and liquid fashion. The powder encompasses a clear polymer with some additive pigments and synthetic fibers to adjust for suitable physical and mechanical properties. The liquid part comprises a methyl methacrylate (MMA, C _5_ O _2_ H _8_ ) monomer, together with cross-linked agents and inhibitors. A synthesis PMMA was reacted once mixing liquid monomer with polymer powder generating polymerization. The reaction is started either by chemical or energy from heat, microwaves, or light to generate a free radical to activate polymerization, and propagated through continuous attaching of monomers until terminating the reaction as the end shifting of free electrons. 13 14 The American Dental Association Specification No. 12 has classified dental polymers into four types: heat-cured, microwave-cured, auto-cured, and light-cured plolymer. 12 Heat-cured polymers are offered in the forms of PMMA powder containing benzoyl peroxide initiator, dibutyl phthalate plasticizer, titanium or zinc oxide opacifiers, fibers, and pigments, while the MMA-liquid part contains ethylene glycol dimethacrylate cross-linked agent and hydroquinone inhibitor. 12 The polymerization reaction begins once the mixing of both components and requires heat energy to activate the benzoyl peroxide initiator, which detaches into carbon dioxide, producing free radicals to activate polymerization. 14 Microwaves are another resource of heat energy that is capable of polymerizing PMMA. However, a nonmetallic container is required for the polymerization process. 15 16 The auto-cured polymer has a distinctive composition that requires a dimethyl- *p* -toluidine initiator to activate the benzyl peroxide in generating free radicals for initiation of the polymerization. Light-cured polymers are offered in a premixed form containing silica fillers, urethane dimethacrylate, and resin monomers, and used a camphorquinone photo-sensitive agent as an initiator to activate and generate free radicals once exposed to light for a specific time to complete polymerization. 15

Flexural strength is a crucial aspect of provisional restorations, especially in the case of long-span restoration with narrow connectors, or in patients having parafunctional habits, clenching, or bruxism. 13 17 18 19 Yet, the use of PMMA for provisional restoration still faces a problem of radiographic imaging since the material is radiolucent and cannot be identified in the radiograph. A radiopaque material is added to the PMMA to allow for visualization of the provisional restoration since it can attenuate the X-rays because it has a higher atomic number and density than the polymeric material. The most commonly used radiopaque material is barium sulfate (B; BaSO _4_ ), which is a white, tasteless, and odorless powder and is regularly used in intestinal radiography. 20 21 22 23 Adding the radiopaque material at an appropriate concentration into PMMA powder may solve the problem of invisible provisional restoration in CBCT imaging. The appropriated radiodensity provisional restoration allows the clinician to visually plan the restoration in conjunction with the osseous structures. Predictable treatment planning can be achieved at this phase and permit other adjunctive treatments such as ridge augmentation or muco-osseous surgery to be performed before implant placement. Some investigations reported the effect of barium sulfate on the mechanical properties of PMMA bone cement used in spine reconstructive surgery. 24 25 26 27 Nevertheless, to the best of the authors' knowledge, there is no evidence reported about the effect of barium sulfate addition to PMMA on the radiodensity and strength of the provisional restorative material. This study aimed to evaluate the radiodensity and flexural strength of provisional restorative material upon adding different compositions of barium sulfate. The null hypothesis was established with no significant differences in radiodensity and flexural strength of the PMMA provisional materials upon different compositional additions of barium sulfate.

## Materials and Methods

The study trailed the CRIS standards for *in vitro* study. The appraised sample size ( *n* ) was calculated according to Eq. 1 by G*power 3.1.9.7 software (Heinrich-Heine University, Dusseldorf, Germany), based on the means ( *µ* ) and standard deviations (s) from the former study 28 at 90% powers of the test; α-error = 0.05, and β *-* error = 0.1.



where standard normal deviation (Z _α_ = 1.96, Z _β_ = 1.28), s = standard deviation (s _1_ = 5.1, s _2_ = 5.4), and µ= mean of tested group (µ _1_ = 78.1, µ _2_ =5.4). The calculated sample size was 15 specimens/group used for this investigation.

### Specimen Preparation

**Fig. 1:**
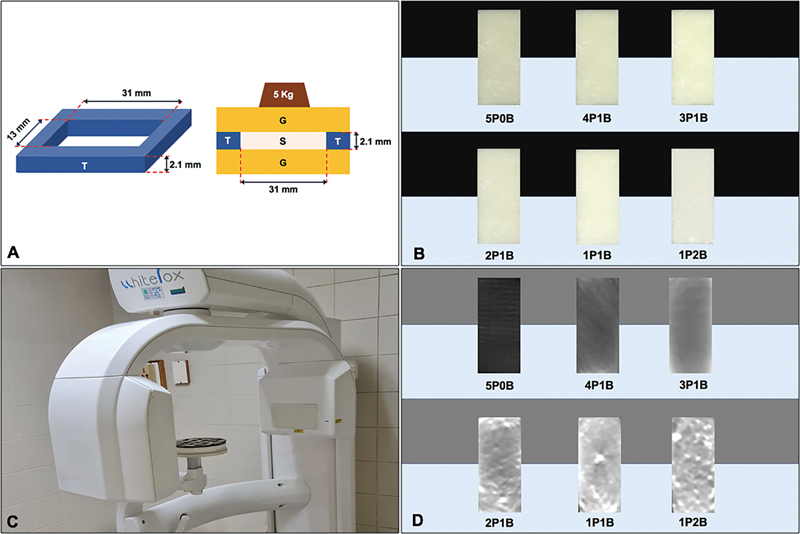
A rectangular shape specimen; S of provisional resins were prepared in the Teflon mold; T and covered with the glass plates; G on the top and bottom of the mold and placed with 5 kg weight on top of the mold assembly **(A)** . The specimens comprised different compositions of polymer; P and barium sulfate; B were precisely prepared **(B)** , radiographically scanned with the cone beam computerized tomography machine **(C)** , and calibrated for radiodensity in the Hounsfield unit for each group **(D)** .

Ninety (90) rectangular shape specimens (length, width, height = × 30 × 12.5 × 2.0 mm) were fabricated complying with the ISO No. 10477–2020 (polymer-based crown and veneering materials). 29 The specimens were prepared at a controlled room temperature of 23 ± 2°C and relative humidity of 30 to 32%. 29 A commercially available conventional auto-polymerized PMMA polymer (Unifast Trad ivory resin, GC, Tokyo, Japan) and barium sulfate (Gammaco, Nonthaburi, Thailand) with particle sizes ranging from 0.4 to 0.9 μm were utilized ( Table 1 ). The PMMA powder of polymer (P) and the powder of barium sulfate (B) were mixed at six different compositions of P/B ratio (each of which contained a different percentage of B) by volume: 5P0B (0%), 4P1B (20%), 3P1B (25%), 2P1B (33.3%), 1P1B (50%), and 1P2B (66.7%), as listed in Table 2 (15 specimens per group). The P and B powders were automatically mixed in a rotational mixer (MajorMix 300, Prominent, Bangkok, Thailand) at 2,900 rpm speed for 10 seconds. The mixed powder was blended with the MMA liquid monomer (Unifast Trad, GC) at a 1:2 volume ratio and thoroughly mixed for 15 seconds to obtain a homogeneous stringy mixture. Then, the mixture was poured and condensed into the preformed polytetrafluoroethylene mold (T, Teflon, Fantastic triumph, Bangkok, Thailand) with specific dimensions (length, width, height = 31 × 13 × 2.1 mm) to compensate for approximately 3.5% polymerization shrinkage of polymer, then covered with the glass plates (G) on the top and bottom of the mold and placed a 5 kg weight on top to squeeze the excess polymer ( Fig. 1A ). All manipulations should be finished within 2 minutes after mixing while the setting starts. The specimens were left for 5 minutes to ensure a complete polymerization process. Then, the G-plates were removed to retrieve the specimen from the mold, and visually examined without magnification at the surface of the specimen without faulty defects, voids, clefts, porosities, or air inclusions; if not, they were discarded. 29 All specimens were polished with aluminous oxide abrasive paper up to grit 5,000 and subsequently polished with diamond suspension (1 μm) solution in a polishing machine (Ecomet-3, Beuhler, Lake Bluff, Illinois, United States) to derive a smooth surface with final specimen dimension ( Fig. 1B ), and kept in the distilled water at 37°C for 24 hours before testing.

**Table 1:** Material, material abbreviation (Abv.), brand/manufacturers, batch number, and composition of materials used in this study

Material	Abv.	Brand/manufacturer	Batch no.	Composition (weight%)
Poly-methyl methacrylate polymer	P	Unifast Trad ivory resin, GC Corp., Tokyo, Japan	2110151	Poly-methyl methacrylate powder, dibenzoyl peroxide <1% w/w, colorant <1% w/w
Methyl methacrylate monomer	M	Unifast Trad Liquid, GC Corp., Tokyo, Japan	2108291	Methyl methacrylate 90–100%, accelerant 1–5%, UV-light absorber <1%, dimethacrylate <0.5%
Barium sulfate (BaSO _4_ )	B	Mahco, Gammaco, Nonthaburi, Thailand	4806178	Barium sulfate nanoparticle

**Table 2:** Mean, standard deviation (SD) of radiodensity in Hounsfield unit (HU), flexural strength (σ, MPa), characteristic strength (σ _0_ , MPa), and Weibull modulus (m) upon different groups of provisional resin comprising different compositions (in ratio and percentage by volume) of powder of polymer; P and barium sulfate; B

Group abbreviation	Ratio		Percentage		n	Mean ± SD			
	P	B	P	B		Hounsfield (Hu)	σ (MPa)	σ _o_ (MPa)	m
5P-0B	5	0	100	0	15	− 283.3 ± 73.8	60.3 ± 15.2	65.9 ± 7.9	4.6 ± 1.9
4P-1B	4	1	80	20	15	731.7 ± 58.7	49.3 ± 6.6	52.1 ± 7.9	8.3 ± 2.0
3P-1B	3	1	75	25	15	748.3 ± 60.2	38.8 ± 7.3	41.8 ± 3.9	5.9 ± 1.1
2P-1B	2	1	66.7	33.3	15	801.6 ± 78.6	33.2 ± 7.4	36.2 ± 6.5	4.9 ± 1.9
1P-1B	1	1	50	50	15	868.3 ± 44.7	19.8 ± 6.1	22.1 ± 2.0	3.4 ± 0.7
1P-2B	1	2	33.3	66.7	15	1,080.2 ± 119.6	15.9 ± 3.4	17.3 ± 5.2	5.2 ± 1.6

### Determination of Radiodensity

The radiographic density of the specimens was accomplished using a three-dimensional CBCT scan (WhiteFox, Acteon, Claudio Giani, Italy). The equipment was set with all necessary elements according to the radiological techniques comprising exposure time of 0.50 second, current of 900 mA, voltage of 105 kV, dose area product of 2,031.43 mGy cm ^2^ , and the focal distance of 65 cm from the specimens ( Fig. 1C ). Specimens in each material group were scanned and their radiodensity measured in HU 6 23 using dental imaging software (Romexis Viewer Version 5.0.R, Planmeca, Helsinki, Finland; Fig. 1D ). The HU values were automatically calculated by the software with its built-in density measuring tool. Each specimen was divided into eight areas (length, width, height = 7.5 × 6.25 × 2.0 mm) to measure radiodensity at the center of each area for each specimen. The average values of HU for each specimen were recorded and further calculated.

### Determination of Flexural Strength

**Fig. 2:**
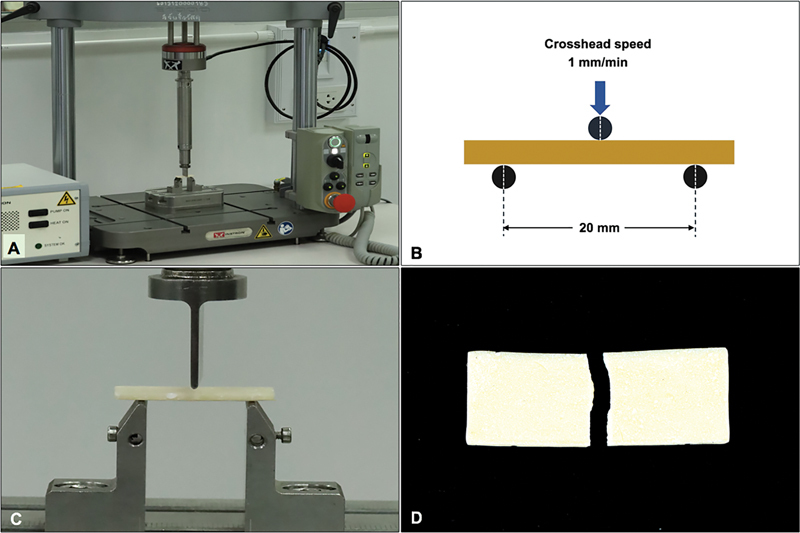
A rectangular shape specimen was placed in a universal testing machine **(A)** on three-point bending test apparatus having the center of the specimen to be at the midpoint between two supporting rollers that placed 20 mm far apart **(B)** , and compressively loaded with the upper roller at a cross-head speed of 1.0 mm/min **(C)** until fracture **(D)** .



The specimens were evaluated for flexure strength using a uniaxial flexural test 29 in a universal testing machine with an autograph (ElectroPuls E1000, Instron, Norton, Massachusetts, United States; Fig. 2A ). The sample was horizontally placed on the three-point flexural test apparatus, by centering the specimen at the midpoint between two supporting rollers that were placed 20 mm apart from each other ( Fig. 2B ). A specimen was compressively loaded with the upper roller at the central part of the specimen at 1.0 mm/min cross-head speed ( Fig. 2C ) until fracture ( Fig. 2D ). The maximum load at fracture (P, Newton) was calculated for flexural strength (σ, MPa) using Eq. 2 .

where *L* = length between the supporting rollers (mm), *W* = specimen width (mm), *D* = specimen height (mm).

### Determination of the Microstructure

Specimens represented as-polymerized and as-fractured surfaces (three specimens each) were randomly selected from each group for microscopic examination. The specimens were cleaned with distilled water, dried in the auto-desiccator at normal ambient temperature for 24 hours, and sputter-coated with gold-palladium in a coating apparatus (K500X, Quorum Technology, Kent, United Kingdom) at 10 mA current and under vacuum (130 mTorr) for 3 minutes, and examined their surface morphology with a scanning electron microscope (SEM; SU3800, Hitachi, Tokyo, Japan) at ×4K magnification as well as chemically characterized with energy dispersive spectroscopy (Oxford, High Wycombe, United Kingdom). The fractured surfaces were analyzed with SEM at ×40 magnification to detect fracture patterns, crack initiation, and defects. 26

### Statistical Analysis

The radiodensity and flexural strength data were accomplished with the Shapiro–Wilk normality test and Levene's homoscedasticity test. An analysis of variance (ANOVA) was applied to justify significant differences in radiodensity and flexural strength using statistical software (SPSS V-28, IBM, Chicago, Illinois, United States). Post-hoc Tukey multiple comparisons were justified for significantly different radiodensity and flexural strength among groups. The significance level for the investigated hypotheses was set at α = 0.05. The reliability of flexural strength was further analyzed for Weibull statistics based on the cumulative probability of survival using Microsoft Excel V-16 (Microsoft, Richmond, Washington, United States), and determined for characteristic strength (σ _o_ ) and Weibull modulus ( *m* ) in concurrence with the slope of the graph sketched between ln{ln(1/ *P*_s_ ( *V*_o_ ))} and *m* ln(σ/σ _o_ ) using Eq. 3 . The association of radiodensity and flexural strength upon different barium sulfate concentrations was analyzed using Spearman correlation analysis.



where *P _s_* ( *V _o_* ): survival probability of sample *,* σ: flexural strength; σ _o_ : characteristic strength, m: Weibull modulus *.*

## Results

**Fig. 3:**
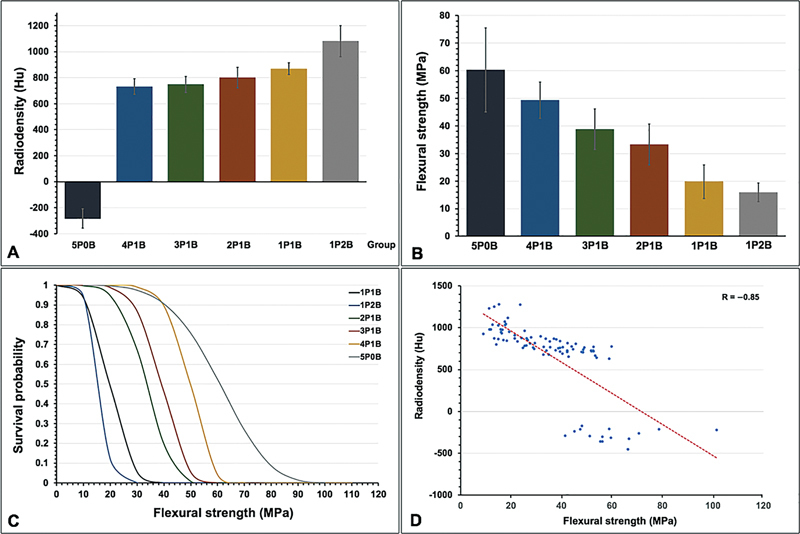
Radiodensity **(A)** and flexural strength **(B)** , Weibull analysis **(C)** , and correlation of radiodensity with flexural strength **(D)** of provisional resins comprising different polymer compositions; P and barium sulfate; B were reported.

The mean and standard deviation (SD) of radiodensity in HU, flexural strength (σ), characteristic strength (σ _o_ ), Weibull modulus ( *m* ), Weibull analysis, and correlation between radiodensity and σ of provisional material comprising different compositions of polymer (P) and barium sulfate (B) are presented ( Table 2 and Fig. 3 ). The mean ± SD of radiodensity (HU) was intense in group 1P-2B (1,080.28 ± 119.8), whereas softest in group 5P-0B (−283.3 ± 73.8). The radiodensity intensely increased upon adding the amount of B from 0, 20, 25, 33.3, 50, and 66.7%, respectively. ANOVA signified a statistically significant difference in radiodensity among the different groups of polymers ( *p* < 0.05; Table 3A and Fig. 3A ). The study indicated that the amount of B comprising in polymer significantly influenced the radiodensity ( *p* < 0.05), as evidenced from one-way ANOVA ( Table 3A ). Post-hoc Tukey multiple comparison tests indicated statistically significant differences in radiodensity among groups of polymers ( *p* < 0.05), except between 4P-1B/3P-1B/2P-1B and 2P-1B/1P-1B ( *p* > 0.05; Table 4A ). Concerning flexural strength, the mean ± SD of σ (MPa) was highest in group 5P-0B (60.3 ± 15.2), whereas lowest in group 1P-2B (15.9 ± 3.4). The σ value decreased upon increasing the amount of B added. ANOVA signified a statistically significant difference of σ among groups of polymers ( *p* < 0.05; Table 3B and Fig. 3B ). The study indicated that the amount of B comprising in polymers significantly influenced σ ( *p* < 0.05), as evidenced from one-way ANOVA ( Table 3B ). Post-hoc Tukey multiple comparisons indicated statistically significant differences in σ among groups of polymers ( *p* < 0.05), except between 3P-1B/2P-1B and 1P-1B/1P-2B ( *p* > 0.05; Table 4B ). Furthermore, significant differences in σ _o_ were indicated among groups of polymers ( *p* < 0.05), except between 3P-1B/2P-1B and 1P-1B/1P-2B ( *p* > 0.05; Table 4B ). The highest *m* -value was observed in group 4P-1B (8.3 ± 2.0), while the lowest *m* -value in group 1P-1B (3.4 ± 0.7). The Weibull analysis of the cumulative survival probability patterns for each test group is displayed in Fig. 3C . The study indicated that increasing the amount of B in polymer resulted in increasing radiographic density, whereas decreasing σ of the polymer, and indicated a negative correlation between radiodensity and σ of the polymer as evidenced from the Spearman correlation coefficient ( *R* ) of −0.83 ( Fig. 3D ).

**Table 3:** ANOVA of (A) radiodensity and (B) flexural strength of various provisional resin materials comprising different compositions of powder of polymer and barium sulfate

A. One-way ANOVA of radiodensity as a function of group of materials					
Source	SS	df	MS	*F*	*p*
Corrected model	17,142,101.867	5	3,428,420.373	587.374	0.001
Intercept	38,943,075.600	1	38,943,075.600	6,671.918	0.001
Material	17,142,101.867	5	3,428,420.373	587.374	0.001
Error	490,296.533	84	5,836.863		
Total	56,575,474.000	90			
Corrected total	17,632,398.400	89			
B. One-way ANOVA of flexural strength as a function of group of materials					
Source	SS	df	MS	*F*	*p*
Corrected model	21,677.907	5	4,335.581	60.024	0.001
Intercept	118,053.118	1	118,053.118	1,634.380	0.001
Material	21,677.907	5	4,335.581	60.024	0.001
Error	6,067.416	84	72.231		
Total	145,798.441	90			
Corrected total	27,745.324	89			

Abbreviations: df, degree of freedom; F, F-ratio; MS, mean square; NB, ; *p* , *p* -value; SS, sum of squares.

**Table 4:** Post-hoc Tukey multiple comparison of (A) radiodensity and (B) flexural strength of various provisional resin materials comprising different compositions of powder of polymer; P and barium sulfate; B

(A) Post-hoc of radiodensity amongst groups of materials comprising different compositions						
Groups	5P-0B	4P-1B	3P-1B	2P-1B	1P-1B	1P-2B
5P-0B	1	0.001	0.001	0.001	0.001	0.001
4P-1B		1	0.991	0.135	0.001	0.001
3P-1B			1	0.402	0.001	0.001
2P-1B				1	0.171	0.001
1P-1B					1	0.001
1P-2B						1
(B) Post-hoc of flexural strength amongst groups of materials comprising different compositions						
Groups	5P-0B	4P-1B	3P-1B	2P-1B	1P-1B	1P-2B
5P-0B	1	0.008	0.001	0.001	0.001	0.001
4P-1B		1	0.014	0.001	0.001	0.001
3P-1B			1	0.476	0.001	0.001
2P-1B				1	0.001	0.001
1P-1B					1	0.813
1P-2B						1

**Fig. 4:**
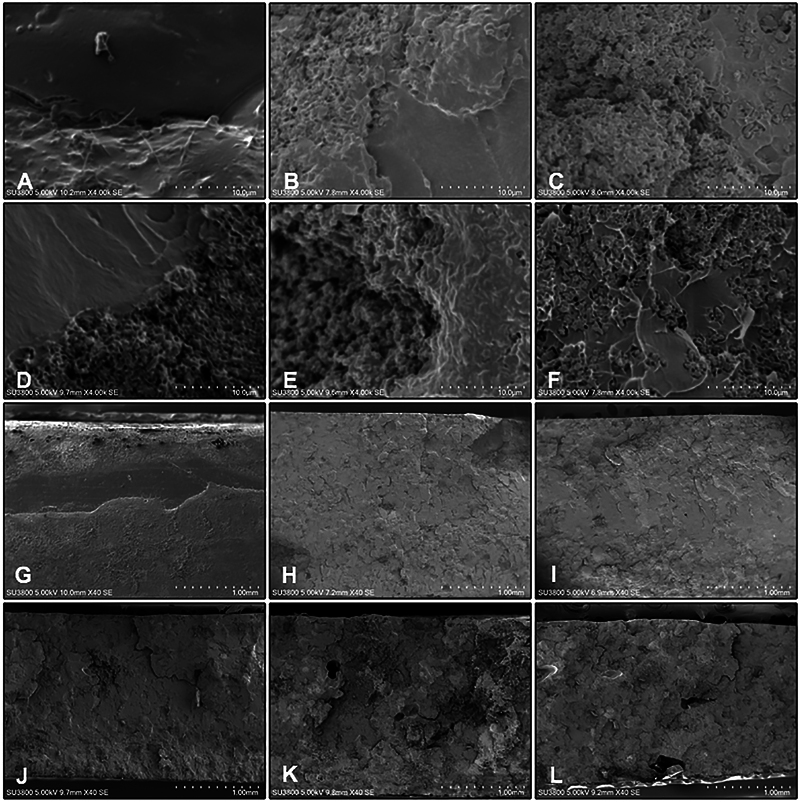
Scanning electron microscope photomicrographs of surfaces at ×4K magnification **(A–F)** and fractography at ×40 magnification **(G–L)** of provisional resins comprising polymer; P and barium sulfate; B at different ratios including 5P0B (A, G), 4P1B (B, H), 3P1B (C, I), 2P1B (D, J), 1P1B (E, K), and 1P2B (F, L). Fractography indicates the origin of fracture, fracture path, and porosity including in the material.

The microstructure for each group was detected by the SEM at ×4K magnification and the surface characteristic structure of the polymer was quantified ( Fig. 4A–F ). The photomicrographs disclosed that the amorphous structure of the polymer was influenced by the distribution of B encircled by acrylate polymer. Several B nanoparticles in uneven surfaces of polymer were noticed as inclusion. The irregular agglomeration of B crystal structures was observed for groups of polymers containing B. Increasing areas of B agglomerates of sub-micron-sized particles were observed as the amount of B contents increased ( Fig. 4B–F ). The SEM photomicrographs at ×40 magnification of the fracture surfaces exposed distinctive characters of fracture surfaces in different groups, as shown in Fig. 4(G–L) , as a result of the different characteristics of materials. The fracture characteristic was a coherent line pattern. The crack lines were fairly straight with slight deviations. The SEM fractography of specimens tested indicated the crack propagation direction from the top toward the bottom. The fractographic analysis indicated that the fracture originated from the loading point of the compressive surface and radially radiated as a fracture path through the tension side of the specimen. For the polymers without the B, cracks departing from pores and propagating in the principal direction are visible ( Fig. 4G ). As B is added, the heterogeneity of the matrix increases, and crack directions are difficult to clarify ( Fig. 4H–L ).

## Discussion

Both radiodensity and strength are the significant properties of restorative materials specifically in restorative reconstrucion. 20 Adequate radiodensity of restorative materials appears white and can certainly be differentiated from the surrounding tissues in diagnostic radiographs, while the strength of restorative material dictates the longevity of the restoration. Due to its polymeric nature, PMMA provisional restoration is a radiolucent material that is hard to detect in the radiographs. It cannot be imaged clearly using the radiographic imaging technique. 14 21 Therefore, the addition of B at different quantities (0, 20, 25, 33.3, 50, and 66.7%) to PMMA provisional material to achieve an appropriate radiographic density and a suitable strength of provisional restoration was the objective of this study. The study revealed that increasing the amount of B powder added to P powder significantly increased radiographic density, but significantly decreased σ of polymer. Thus, the null hypotheses were rejected for both radiodensity and flexural strength upon different compositional additions of B. The nanoparticles of B tend to fill in the molecule of the polymer by being incorporated into the pores or air bubbles in the polymer chain during the mixing process, causing the light to hardly transmit through the translucence polymer molecule, thus intensifying the radiodensity of the material. The increasing radiodensity of the material is owing to the existence of the radiopaque B powder in the polymer that absorbs more radiation than the polymer matrix and shows more radiopacity. This is probably associated with the atomic number of B particles being higher than the chemical constituent of P, which has a low atomic number. The barium atom is large and heavy, thus it absorbs X-rays relatively well as supported by another study. 22 The radiodensity of the materials in this study can be classified into three categories: low (≤350 HU), medium (350–850 HU), and high (>850 HU). 1 23 Radiodensity values (HU) of provisional polymer tested were significantly influenced by the amount of B. Adding B up to 33% to P can produce a medium radiodensity provisional material, which is equivalent to the bone mineral density of D3 according to Misch's classification, whereas adding B higher than 50% can produce a high radiodensity provisional material, which is equivalent to the bone mineral density of D2 according to Misch's classification. 1 It is remarkable that the HU value increases >1,000 as the B concentration is increased by 66.7%. Vice versa, the radiodensity of the self-polymerizing PMMA provisional material for fixed prosthesis was lower than those of the human and bovine dental hard tissues, as supported by other studies. 28 The radiodensity of provisional materials in this experiment complies with the minimum requirements of ISO regulations. 29 However, other mixing ratios of the material need to be further investigated to provide better properties for long-term use.

Concerning flexural strength, it is the only one of several factors affecting the success of a provisional restoration and plays a critical role in restorative dentistry, particularly the patients having parafunctional habits, clenching, or bruxism. 12 18 The present study indicated that the flexural strength of polymer significantly decreased following the increasing additional quantities of B powder. The result was supported by the previous studies that confirmed the negative effect of B on flexural strength. 19 25 This effect is probably because the inorganic nanoparticles of B exist as inclusions within the amorphous structure of the polymer as evidenced by the SEM. There is no adhesion between B and the cured PMMA, which serves as the matrix. Therefore, B nanoparticles agglomerated within the matrix polymer were considered as inclusions, which might play a role in crack nucleation, which possibly triggers the breakdown of the interfaces between the B nanoparticles and polymer matrix as supported by previous studies. 26 27 Once there is a breakdown of this interface, the stress developed under load will not be effectively distributed throughout the material. 22 It is also perhaps due to voids inside the specimens, as well as the edge defects that result in decreasing flexural strength. 13 16 There are no published articles regarding the exact mechanical properties that may best aid the clinician in predicting the *in vivo* performance of provisional materials. 15 Although the flexural strength of PMMA was decreased upon B addition, however, adding B up to 33.3% still fulfilled with the requirement of ISO 10477 guidelines. 29

The present study indicated that the addition of barium sulfate is a useful method for increasing the radiodensity of PMMA as a provisional restorative material and still satisfies an optimal flexural strength. Adding barium sulfate up to 25% of the polymer achieves adequate radiopacity and strength of PMMA for provisional restoration. However, adding more than 33.7% provides better radiographic visibility, but restricts its uses, suggesting for fabrication provisional restoration in a minimal loading area and other appliances, for example, radiographic stent and surgical guide. Mysteriously, the effect of adding barium sulfate on the surface roughness of polymer should be further investigated since it probably affects the bacterial aggregation on the surface of provisional restoration. Regarding the safety of barium in humans, the Department of Health and Human Services and the International Agency for Research on Cancer have not classified barium as carcinogenic. Besides, barium sulfate is medically used as a radiocontrast agent for radiographic imaging and other diagnostic procedures in the esophagus, stomach, and bowels as it is not absorbed by the digestive tract. 30 Nevertheless, clinical studies are needed to determine the optimal P/B ratio to achieve good clinical results and prevent subsequent fracture of provisional restoration.

## Conclusion

In this well-control *in vitro* study, it could be concluded that the radiodensity and strength of provisional restorative materials were influenced by barium sulfate addition to PMMA. Increasing the amount of barium sulfate added to PMMA material significantly increases radiodensity but decreases the flexural strength of provisional restorative material. The study suggested that adding barium sulfate up to 25% provides adequate radiodensity and strength for PMMA provisional restoration, which is possibly better used in high-stress areas, especially the posterior region of the arch or long-span fixed provisional restoration where extreme strength material is required. However, adding barium sulfate of more than 33.7% provides better radiographic visibility of the polymer, probably suggested for construction of a less extensive provisional restoration at less-stress regions and other appliances such as radiographic stent and surgical template for accurate implant planning and placement, which require not as much of strength.

## Clinical Implications

To produce provisional restoration with appropriate radiodensity and optimal strength, it is recommended to add barium sulfate, up to 25%, into PMMA provisional material, which is suitable for the fabrication of intense radiodensity and optimal strength provisional restoration, for use in high-stress areas or for long-span fixed provisional restoration. Addition of barium sulfate more than 33.7% provides better radiographic clarification, but compromises the strength of PMMA, suggesting for construction of a less extensive provisional restoration at less-stress regions and other appliances that do not require much strength such as radiographic stent and surgical template.
